# A unique homologue of the eukaryotic protein-modifier ubiquitin present in the bacterium *Bacteroides fragilis*, a predominant resident of the human gastrointestinal tract

**DOI:** 10.1099/mic.0.049940-0

**Published:** 2011-11

**Authors:** Sheila Patrick, Kelly L. Jobling, Danny O’Connor, Zubin Thacker, David T. F. Dryden, Garry W. Blakely

**Affiliations:** 1 Centre for Infection and Immunity, School of Medicine, Dentistry and Biomedical Sciences, Queen’s University Belfast, Medical Biology Centre, Belfast BT9 7BL, UK; 2 Institute of Cell Biology, University of Edinburgh, Darwin Building, The Kings Buildings, Edinburgh EH9 3JR, UK; 3 EaStChem School of Chemistry, University of Edinburgh, The Kings Buildings, Edinburgh EH9 3JJ, UK

## Abstract

In the complete genome sequences of *Bacteroides fragilis* NCTC9343 and 638R, we have discovered a gene, *ubb,* the product of which has 63 % identity to human ubiquitin and cross-reacts with antibodies raised against bovine ubiquitin. The sequence of *ubb* is closest in identity (76 %) to the ubiquitin gene from a migratory grasshopper entomopoxvirus, suggesting acquisition by inter-kingdom horizontal gene transfer. We have screened clinical isolates of *B. fragilis* from diverse geographical regions and found that *ubb* is present in some, but not all, strains. The gene is transcribed and the mRNA is translated in *B. fragilis*, but deletion of *ubb* did not have a detrimental effect on growth. BfUbb has a predicted signal sequence; both full-length and processed forms were detected in whole-cell extracts, while the processed form was found in concentrated culture supernatants. Purified recombinant BfUbb inhibited *in vitro* ubiquitination and was able to covalently bind the human E1 activating enzyme, suggesting it could act as a suicide substrate *in vivo*. *B. fragilis* is one of the predominant members of the normal human gastrointestinal microbiota with estimates of up to >10^11^ cells per g faeces by culture. These data indicate that the gastro-intestinal tract of some individuals could contain a significant amount of aberrant ubiquitin with the potential to inappropriately activate the host immune system and/or interfere with eukaryotic ubiquitin activity. This discovery could have profound implications in relation to our understanding of human diseases such as inflammatory bowel and autoimmune diseases.

## Introduction

The highly conserved protein-modifier ubiquitin has, to date, only been found in eukaryotes, where tagging of proteins with ubiquitin (ubiquitination) is intrinsic to control of diverse processes central to cell function. The first role identified for ubiquitin was the regulation of intracellular proteolysis via the 26S proteosome, in which ubiquitin becomes covalently bound to a substrate that is subsequently targeted for degradation (Hochstrasser, 2009). The first step of ubiquitination in eukaryotes is the covalent attachment of ubiquitin to the cognate activating enzyme E1, initially by adenylation of the C terminus of ubiquitin followed by formation of a thioester bond with the active site cysteine of E1. The covalently attached ubiquitin is subsequently transferred to the active site cysteine of the ubiquitin-conjugating enzyme E2, followed by formation of an isopeptide bond with the final substrate mediated by the ubiquitin ligase enzyme E3 (Komander, 2009).

It is now recognized that post-translational regulation by ubiquitination also plays an important role in modification of protein function, including cell cycle progression, membrane protein endocytosis, intracellular trafficking, ribosome biogenesis, signal transduction, DNA repair, stress responses, chromatin-mediated regulation of transcription and antigen presentation.

Ubiquitin is also a major factor involved in development and function of both the innate and adaptive immune systems. Mis-regulation of the ubiquitin pathway is therefore implicated in a wide range of diseases, including cancer, cardiac disease, neurodegenerative disorders and type 2 diabetes (Schwartz & Ciechanover, 2009; Rodríguez *et al.*, 2009; Komatsu & Ichimura, 2010; Vereecke *et al.*, 2009). Immune surveillance for invading pathogens is also controlled by ubiquitin. Extracellular pathogens are recognized by Toll-like receptors that activate a cascade leading to phosphorylation of IκBα, the inhibitor of NF-κB. Ubiquitination and subsequent proteolytic degradation of IκBα releases NF-κB for entry into the nucleus where it activates transcription of pro-inflammatory associated genes (Karin & Ben-Neriah, 2000). Similarly, regulatory events triggered by nucleotide oligomerization domain-like receptor recognition of intracellular pathogens involve ubiquitination (Shaw *et al.*, 2008). In addition, *Salmonella enterica* internalized by macrophages is recognized by a cytosolic mechanism that stimulates direct polyubiquitination of bacterial surface proteins with subsequent recruitment of the proteosome, followed by destruction of the invading prokaryote and presentation of derived peptides to class I major histocompatibility complex molecules (Perrin *et al.*, 2004).

While the evolutionary origin of eukaryotic ubiquitin is hypothesized to be rooted within a common prokaryotic ancestor, structural homologues of ubiquitin identified in bacteria do not have sequence similarity, apart from two terminal glycine residues; examples include the MoaD and ThiS proteins in *Escherichia coli* involved in thiamine and molybdopterin biosynthesis, respectively. These proteins are not involved in proteolysis but do utilize chemistry similar to ubiquitin conjugation, i.e. formation of a thioester bond between the C-terminal glycine and a cysteine catalytic residue in a complementary activating enzyme (Hochstrasser, 2009).

The primary mechanisms for regulation of protein half-life and translation quality control in bacteria do not involve homologues of eukaryotic ubiquitin. In *E. coli* the N-terminal rule pathway dictates the stability of a polypeptide via an adaptor protein, ClpS, which targets degradation by the ClpAP protease (Erbse *et al.*, 2006). Prematurely terminated translation products are marked at the C terminus with an 11 amino acid SsrA tag that directs the polypeptide for degradation by either ClpXP or ClpAP (Gottesman *et al.*, 1998). The analogous N-terminal rule pathway in eukaryotes uses the specificity of a class of ubiquitin E3 ligases, the N-recognins, to direct proteolysis by the proteosome (Tasaki *et al.*, 2005). A protein-tagging system that functions in a similar manner to the ubquitin–proteosome pathway has been identified in *Mycobacterium tuberculosis*; however, the Pup protein that becomes covalently attached to proteins destined for digestion does not share sequence homology with eukaryotic ubiquitin (Pearce *et al.*, 2008).

We now report evidence of horizontal gene transfer from a eukaryotic source into a bacterium of the normal human resident microbiota which results in expression of a secreted protein with 63 % identity to human ubiquitin. The Bacteroidetes are predominant members of the gastro-intestinal (GI) tract microbiota. By culture, *B. fragilis* represents ~10–15 % of the members of the *Bacteroides* present in faeces, with estimates between 10^11^ and 10^12^ cells g^−1^ (Patrick, 2002). *B. fragilis* is also the most frequently isolated obligately anaerobic Gram-negative bacterium from life-threatening human infections, such as intra-abdominal, vaginal and brain abscesses (Patrick, 2002; Patrick & Duerden, 2006), and is a major cause of anaerobic bacteraemia, with a potential mortality rate of up to ~30 % (Cheng *et al.*, 2009). Such infections generally arise from faecal contamination of normally uncolonized body sites, for example, peritonitis following perforation of the bowel (Patrick & Duerden, 2006). The discovery that ubiquitin-positive *B. fragilis* is present in the GI tract, however, could have profound implications for our understanding of several human diseases in which ubiquitin malfunction is implicated, and indeed also in relation to the development of autoimmune disease.

## Methods

### 

#### Bacterial strains and growth conditions.

The isolates used in this study were *B. fragilis* NCTC9343, NCTC9344 and NCTC10584; *Bacteroides ovatus* Queen’s University Belfast culture collection; and clinical isolates obtained from Craigavon Area Hospital Northern Ireland (designated ‘LS’), the Royal Victoria Hospital Northern Ireland (designated ‘JC’), the Free University of Amsterdam kindly supplied by J. van Doorn (designated ‘BE’), the University of Edinburgh Scotland kindly supplied by I. Poxton (designated ‘GNAB’); and a rifampicin-resistant mutant of an isolate from Chigaco, USA, kindly supplied by C. J. Smith (638R). Identification was confirmed with the API 20A system or PCR and sequencing. All *Bacteroides* strains were grown in an anaerobic atmosphere containing 10 % CO_2_, 10 % H_2_ and 80 % N_2_ in either supplemented brain heart infusion broth or defined medium (DM) (Van Tassell & Wilkins, 1978).

#### Molecular and immunological techniques.

*ubb* from genomic DNA was amplified by PCR from *Bacteroides* strains using *Pfu* polymerase (NEB) using *ubb*-specific oligonucleotide primers designed using the NCTC9343 DNA sequence. RNA was isolated from cultures grown in DM (samples taken at OD_600_ 0.4, 0.8 and 1.6) using the Qiagen RNeasy kit and quantified using a Hitachi U-2000 spectrophotometer. RT-PCR analysis of RNA samples used the Qiagen Omniscript RT kit with 20 ng RNA template. The absence of DNA contamination in RNA samples was confirmed by omitting the reverse transcription step and performing the PCR amplification. DNA was analysed by electrophoresis through 1 % agarose gels followed by staining with ethidium bromide and visualization by UV light. The *ubb* deletion mutant was generated in NCTC9343 by replacing *ubb* with an erythromycin resistance cassette using our previously described method (Patrick *et al.*, 2009).

The *ubb* gene was cloned as a 6×His-tagged N-terminal fusion in pTRC99a. Recombinant (r)BfUbb was purified to homogeneity by overexpression in *E. coli* DH5α, followed by affinity purification using Qiagen Ni-NTA agarose resin and gel filtration through a Superose 12 10/300 GL column (GE Healthcare). Polyclonal antiserum was produced by inoculation of a New Zealand white rabbit with rBfUbb conjugated to keyhole limpet haemocyanin (KLH) carrier protein using an Imject Immunogen EDC kit with mcKLH (Thermo Scientific). The conjugated antigen was suspended in PBS containing QuilA (0.125 mg ml^−1^) adjuvant (Brenntag). rBfUbb (0.2 mg) was inoculated at approximately 4 week intervals over a 6 month period under UK Government Home Office Personal and Project Licences and with local ethical approval. Rabbit anti-bovine ubiquitin was obtained from Sigma-Aldrich. Whole cells for immunoblots were electrophoresed through 12 % polyacrylamide gels, transferred to a PVDF membrane and incubated with the appropriate primary and secondary antibodies before detection using the ECL+ chemiluminescent system (GE Healthcare). Supernatants from cultures grown in DM were prepared by centrifugation, filtration through a 0.45 µm membrane and concentrated by centrifugation through a membrane with a 100 kDa cut-off (Amicon Ultra; Millipore). Concentrated supernatant samples for immunoblotting were separated by electrophoresis through 16 % SDS Tris-Tricine polyacrylamide gels. Immunoblots were incubated with the appropriate primary antibody followed by anti-rabbit alkaline phosphate antibody conjugate and reacted with Sigma *Fast* BCIP/NBT(Sigma). Bacterial genome sequences were viewed with Artemis and the Artemis Comparison Tool (Carver *et al.*, 2008).

#### *In vitro* ubiquitination.

*In vitro* ubiquitination reactions were carried out in 25 mM HEPES and 0.5 mM DTT at 37 °C for 180 min and used the fraction II HeLa conjugation kit (Boston Biochemicals) with a biotinylated lysozyme substrate (Boston Biochemicals). Reactions contained 4 mg Fraction II HeLa ml^−1^, 2.5 mg ubiquitin ml^−1^, 4 µM ubiquitin aldehyde, 5 µM MG-132 and an ATP regeneration system. Reactions containing 280 µg BfUbb ml^−1^ were pre-incubated at 37 °C for 15 min prior to the addition of ubiquitin. Protein samples were separated by electrophoresis through 12 % polyacrylamide gels followed by transfer to a PVDF membrane and detection of biotinylated lysozyme using an avidin–HRP conjugate (Boston Biochemicals). Binding of BfUbb to human E1 was carried out in reactions containing 390 µg BfUbb ml^−1^ and either 55 µg or 27.5 µg E1 ml^−1^ (Boston Biochemicals) in 50 mM HEPES pH 8.0 at 37 °C for 60 min. Samples were analysed on a non-reducing 12 % polyacrylamide gel followed by transfer to a PVDF membrane and detection with polyclonal anti-eukaryotic ubiquitin antibodies (Chemicon) and the ECL+ chemiluminescent system (GE Healthcare).

## Results

### A horizontally acquired gene encoding ubiquitin in *B. fragilis*

**Fig. 1.**
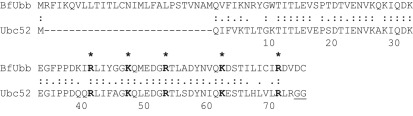
A homologue of ubiquitin present in the bacterium *B. fragilis*. Alignment of the predicted polypeptide encoded by the *ubb* gene in *B. fragilis* with human ubiquitin Ubc52. The first 28 residues of BfUbb represent the putative signal sequence. Identical and similar amino acids are indicated, with residues important for interactions with E1 activating enzyme shown in bold and with asterisks. The two C-terminal glycine residues of Ubc52 involved in covalent attachment to the E1-activating enzyme are underlined.

The annotation of the *B. fragilis* NCTC9343 genome indicates that it encodes a number of polypeptides with similarities to human proteins (Cerdeño-Tárraga *et al.*, 2005). The most striking homology, however, is found with a putative protein encoded by BF3883 that has 63 % identity (48 of 76 amino acids) to human ubiquitin, such as Uba52 or UbcEP2 (Fig. 1). While many of the amino acids important for interactions with the E1 activating enzyme are conserved in *B. fragilis* ubiquitin (BfUbb), it has at least two unusual features. Firstly, it contains a signal sequence and secondly, it does not possess the two terminal glycine residues required for covalent interactions with the E1 and E2 enzymes of the ubiquitin pathway (Fig. 1).

Despite the implication of its name, ubiquitin has to date only been found in eukaryotes. BF3883 is contained within an 11 kb sequence exhibiting a low GC content, with the closest DNA homology to the gene being 76 % identity (151/199 bp; E = 5×10^−29^) to a ubiquitin-encoding sequence, MSV144, from a migratory grasshopper entomopoxvirus (GenBank accession no. AF063866.1). This suggests that BF3883 was acquired by inter-kingdom horizontal gene transfer. Hereafter, we refer to BF3883 as *ubb*.

**Fig. 2.**
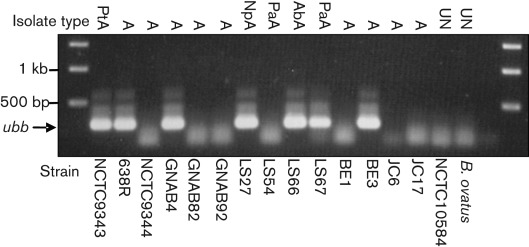
Agarose gel showing detection of the *ubb* gene by PCR in isolates of *B. fragilis* from different geographical locations. Origins of *B. fragilis* samples are: PtA, peritoneal abscess; A, abscess; NpA, neoplasm abscess; PaA, perianal abscess; AbA, abdominal abscess; UN, unknown origin. The position of the *ubb* amplicon is shown adjacent to the image, with DNA molecular size markers in the first and last lanes. Note the absence of *ubb* in *B. ovatus* and some *B. fragilis* isolates.

The genome sequences of two other *B. fragilis* strains, 638R (isolated in the USA) and YCH46 (isolated in Japan), have also been determined (Patrick *et al.*, 2010; Kuwahara *et al.*, 2004). Strain 638R contains a similar low GC region that includes a putative ubiquitin-encoding gene (BF638R3923) which has 100 % sequence identity to *ubb*. In contrast, the YCH46 genome only has three genes from the equivalent low GC region and does not contain *ubb*, suggesting that the ubiquitin gene has been deleted. To determine the prevalence of *ubb* in other *B. fragilis* strains, we screened a number of isolates derived from different source materials and different geographical locations. Analysis by PCR demonstrated that *ubb* was present in clinical isolates from patients in Edinburgh, Belfast and Amsterdam (Fig. 2). Sequence comparison of the *ubb* amplicons with the NCTC9343 sequence showed 100 % conservation within the coding region (data not shown). While there was no obvious correlation with the presence or absence of the ubiquitin gene and the source of the sample, this demonstrates that the *ubb* gene is present in *B. fragilis* strains resident in the GI tract of individuals from diverse populations. Homologues of *ubb* were not found in other *Bacteroides* species, by either PCR or genome sequence analysis.

To determine whether BfUbb has a central role in the metabolism of *B. fragilis* NCTC9343, as YCH46 which lacks *ubb* has a slower growth rate and attains lower cell density in stationary phase (Patrick *et al.*, 2010), we generated a strain in which *ubb* was deleted. Using our previously described method, *ubb* was replaced with an erythromycin resistance cassette (Patrick *et al.*, 2009). The resulting Δ*ubb* : : *ermF* strain did not show any cell morphology defects and had the same growth rates compared with the parental strain in BHI-S medium and glucose defined medium (DM). This indicates that BfUbb is not essential for *B. fragilis* growth in laboratory medium, although it may be advantageous for survival within the host.

### Expression of BfUbb in *B. fragilis*

**Fig. 3.**
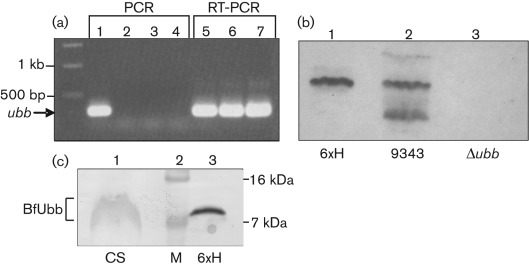
Detecting expression of BfUbb. (a) Agarose gel showing transcription of *ubb* detected by RT-PCR. Lanes: 1, PCR using genomic DNA; 2–4, PCR using RNA from cultures at OD_600_ 0.4, 0.8 and 1.6 (indicates absence of DNA contamination); 5–7, RT-PCR using RNA from cultures at OD_600_ 0.4, 0.8 and 1.6. The position of *ubb* is indicated with an arrow. (b) Immunoblot using rabbit anti-BfUbb polyclonal antiserum. Lanes: 1, purified 6×His-BfUbb; 2, whole-cell extract of *B. fragilis* NCTC9343; 3, whole-cell extract of *B. fragilis* Δ*ubb*. The lowest band in lane 2 represents a degradation product of BfUbb. (c) Immunoblot of concentrated supernatant from a culture of NCTC9343 grown in DM (lane 1), compared with purified 6×His-BfUbb (lane 3). Molecular mass markers are shown in lane 2.

Since it is possible that *ubb* had been acquired from a eukaryotic source, the gene may be an evolutionary relic and may or may not be integrated adjacent to a promoter. We confirmed active transcription of *ubb* in *B. fragilis* by detection of mRNA using RT-PCR (Fig. 3a). Total RNA was isolated from cultures grown in DM during early exponential growth and upon entry into stationary phase. RT-PCR products were detected from RNA templates derived from both growth phases, suggesting that *ubb* is transcribed by the housekeeping RNA polymerase of *B. fragilis*.

We cloned and expressed the predicted processed form of BfUbb as a 6×His-tagged fusion in *E. coli* and used this protein to generate rabbit polyclonal antiserum. Immunoblotting of cell extracts from cultures grown in glucose DM revealed BfUbb as a major protein band of ~9 kDa, plus a second band of ~12 kDa (Fig. 3b). This pattern is consistent with cleavage of the signal peptide having generated the abundant form of BfUbb and the ~12 kDa band, representing the holo-protein prior to export and processing. In addition, some lower-molecular-mass bands, interpreted as degradation products were observed. Interestingly, anti-bovine ubiquitin antibodies cross-reacted with purified recombinant 6×His-BfUbb, but did not detect BfUbb in whole-cell extracts of *B. fragilis*. The presence of a signal sequence and its apparent cleavage is consistent with BfUbb being transported to the periplasm of *B. fragilis*. To interact with the eukaryotic host, however, BfUbb would need to be secreted by the bacterium. *B. fragilis* is known to produce outer membrane vesicles (OMVs) that bleb from the cell surface (Patrick *et al.*, 1996). To determine whether BfUbb was potentially present in OMVs, we concentrated supernatants by filtration through membranes with a 100 kDa cut-off. Immunoblotting of these concentrated supernatants showed the presence of a smeared band of BfUbb that migrated close to the position of the purified 6×His-BfUbb (Fig. 3c; note these samples were separated on a higher percentage gel than that used in Fig. 3b). This band was not detected in concentrated supernatants from the Δ*ubb* strain (data not shown). Smeared bands are often characteristic of glycosylated proteins. Two triplet amino acid sequences (DTV and DST; Fig. 1) corresponding to the consensus recognition site for *B. fragilis* *O*-glycosylation (Fletcher *et al.*, 2011) are present in BfUbb. The potential glycosylation of BfUbb is being investigated.

### *In vitro* activity of BfUbb

**Fig. 4.**
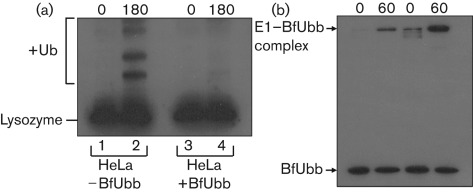
*In vitro* activity of BfUbb. (a) Immunoblot of *in vitro* ubiquitination of lysozyme using HeLa cell extract in the absence (lanes 1 and 2) or presence (lanes 3 and 4) of BfUbb. Samples were taken at the start of the reaction (0) and at 180 min (180). Lane 2 shows the increase in molecular mass of the lysozyme substrate following covalent attachment of ubiquitin (+Ub). This covalent modification is inhibited by the addition of BfUbb (lane 4). (b) Covalent complexes between human E1 and BfUbb, under non-reducing conditions, were detected by immunoblotting using anti-bovine ubiquitin polyclonal serum. Two reactions using different concentrations of E1 are shown, with time points at 0 and 60 min. Note there was some antibody cross-reactivity with E1 at the higher concentration.

Since *ubb* is not present in all strains of *B. fragilis*, and deletion of the gene in NCTC9343 did not have an apparent effect on growth of the bacterium, we hypothesized that BfUbb might be a candidate protein for interaction with the host. To examine the effect of BfUbb on the ubiquitination pathway, we reconstituted the reaction using HeLa cell extracts (Fraction II) and a biotinylated lysozyme substrate. Pre-incubation of purified 6×His-BfUbb with the HeLa extract, prior to addition of a 10-fold excess of eukaryotic ubiquitin, inhibited subsequent covalent attachment of ubiquitin to the substrate (Fig. 4a). This suggested that 6×His-BfUbb was binding to the activating and conjugating enzymes of the ubiquitin pathway and inhibiting their catalytic functions. The first step of ubiquitination is the covalent attachment of ubiquitin to the E1 activating enzyme by formation of a thioester bond with the active site cysteine. The ubiquitin homologues encoded by all *B. fragilis* strains tested do not contain a C-terminal glycine but do have a C-terminal cysteine (Fig. 1). We hypothesized that BfUbb might form a disulphide bridge with human E1 that would lead to its inactivation. Under non-reducing conditions a covalent complex could be detected between 6×His-BfUbb and human E1 by immunoblotting with anti-bovine ubiquitin antibodies (Fig. 4b). This complex was not detected under reducing conditions (data not shown). These data indicated that the two proteins are capable of forming a covalent intermediate in the absence of both ATP and a terminal glycine residue, and suggest that BfUbb could act as a suicide substrate leading to inactivation of E1 and blocking of the ubiquitination cascade.

## Discussion

The presence of *ubb* only in selected strains of *B. fragilis*, the similarity of BfUbb to human ubiquitin and its antigenic cross-reactivity, as well as the potential for BfUbb to interfere with the eukaryotic ubiquitination pathway could have profound implications for a range of human diseases. By culturing faeces, *Bacteroides* species numbers range from 10^9^ to 10^14^ cells g^−1^, of which *B. fragilis* estimates range from 4 to 14 % (reviewed by Patrick, 2002). This provides a potentially large reservoir of an aberrant form of ubiquitin in some humans. The resident GI tract microbiota has a well-proven role in the normal development of the immune system. In addition, inflammatory bowel disease is recognized as being caused by a deficient or abnormal mucosal immune response in individuals who are genetically susceptible, clearly related to the presence of intestinal microbes (Marks *et al.*, 2010; Xavier & Podolsky, 2007). In microscopic studies of the spatial organization of the adherent mucosal microbiota, *B. fragilis* was consistently associated with the mucosal surface of patients with inflammatory bowel disease. It was the predominant member of the adherent mucosal biofilm, accounting for >60 % of the biofilm mass (Swidsinski *et al.*, 2005). It will be intriguing to determine whether, given the intimate association of *B. fragilis* with the human GI tract, BfUbb plays a role in the development of these diseases. Furthermore, we postulate that BfUbb could be a driver of other autoimmune diseases, as a result of generating an autoimmune reaction to either or both ubiquitin or proteins to which ubiqutin is bound.

The genome of *B. fragilis* does not encode the molecular components required to make a proteosome equivalent to the eukaryotic 26S proteosome; nor does it appear to contain the activating and conjugating enzymes of the ubiquitination pathway. The *ubb* gene has evolved from the eukaryotic equivalent to encode a protein containing a bacterial signal sequence predicted to specify export to the periplasm; also, the C-terminal glycine residues that are required for successful ubiquitination of target proteins in eukaryotes are absent. This suggests that BfUbb has been subverted by the bacterium to fulfil a unique role. So what is the function of BfUbb? The high degree of homology between BfUbb and human ubiquitin, with conservation of residues important for interaction with the E1-activating and E2-conjugating enzymes, implies that these interactions are important. Although we have shown that BfUbb can inhibit ubiquitination *in vitro*, it is possible that the C-terminal cysteine residue could form a thiol-ester bond with other amino acids on selected targets that lead to modified protein function. The isopeptide bond formed between eukaryotic ubiquitin and lysine residues is not the only way that conjugates can be generated; for example, the viral E3 ligase MIR1 can catalyse the formation of thiol-ester bonds between ubiquitin and cysteine residues on major histocompatibility complex I class proteins found on the surface of cytotoxic T lymphocytes (Cadwell & Coscoy, 2005).

The periplasmic localization of BfUbb would provide a means for mobilizing the protein into the environment. *B. fragilis* produces a large number of OMVs (Patrick *et al.*, 1996) that are free to diffuse throughout the gut lumen. The detection of BfUbb in supernatants that had been concentrated through a membrane with a 100 kDa cut-off suggests that the protein is associated with extra-cellular macromolecular complexes. OMVs are ‘secretory vehicles’ that can deliver bacterial components to host cells and tissues as well as other bacteria (Kuehn & Kesty, 2005; Bomberger *et al.*, 2009). The OMVs of *B. fragilis* agglutinate erythrocytes, via sodium periodate sensitive agglutinins, and are also known to contain degradative enzymes (Patrick *et al.*, 1996). The presence of unusual sphingolipids in the *B. fragilis* outer membrane may allow fusion between OMVs and eukaryotic cytoplasmic membranes that contain sphingolipids. Alternatively, the vesicles may enter epithelial cells by endocytosis. Ubiquitin plays a central role in the regulation of the pro-inflammatory response. Following exposure to a bacterial pathogen, a Toll-like receptor-initiated signalling cascade ultimately results in the phosphorylation of IκB that is subsequently ubiquitinated and degraded by the proteosome. Destruction of IκB releases NF-κB to enable its nuclear localization and initiation of transcription (Bhoj & Chen, 2009). If BfUbb were to enter the cytoplasm of an epithelial cell and act as a suicide substrate for ubiquitination, it could potentially inhibit activation of NF-κB, thus downregulating the inflammatory response.

BfUbb could also aid the success of *B. fragilis* as an opportunistic pathogen. Many pathogenic bacteria have evolved the ability to avoid or suppress the host immune response by interfering with the ubiquitin-proteosome pathway, although none of these mechanisms involve ubiquitin homologues (Boyer & Lemichez, 2004). For example, *Yersinia pestis* uses YopJ to prevent activation of the innate immune response, *Salmonella enterica* serovar Typhimurium produces a ubiquitin ligase and a deubiquitinating protease that are secreted into infected macrophages, while intracellular *Chlamydia trachomatis* secretes two proteases that have deubiquitinating activities (Zhang *et al.*, 2006; Rytkönen *et al.*, 2007; Le Negrate *et al.*, 2008).

This discovery of a novel ubiquitin homologue in a bacterium, which is a key member of the normal human resident microbiota, could fundamentally alter our understanding of diseases that currently have an unknown aetiology. While we postulate a possible role in inflammatory bowel and autoimmune diseases, mis-regulation of ubiquitin is implicated in a wide range of diseases including cancer, neurodegenerative disorders and type 2 diabetes. The extent to which BfUbb may or may not be involved in any of these diseases remains to be determined, and will require careful consideration of BfUbb-positive *B. fragilis* colonization, the control of BfUbb expression and underlying human genetic predispositions.
